# PI3Kβ Inhibitor TGX221 Selectively Inhibits Renal Cell Carcinoma Cells with Both VHL and SETD2 mutations and Links Multiple Pathways

**DOI:** 10.1038/srep09465

**Published:** 2015-04-08

**Authors:** Chenchen Feng, Yang Sun, Guanxiong Ding, Zhong Wu, Haowen Jiang, Lujia Wang, Qiang Ding, Hui Wen

**Affiliations:** 1 Department of Urology, Huashan Urology, Fudan University, Shanghai 200040, PR China; 2 Brigham and Women's Hospital, Harvard Medical School, MA 02115, USA

## Abstract

We aimed to exploit novel compounds with high selectivity to clear cell renal cell carcinoma (ccRCC) with common mutations. Using the GDSC databases, we searched for compounds with high selectivity for ccRCC with VHL and/or SETD2 mutations. Clinical impact and gene interactions were analysed using TCGA database. In vitro and in vivo studies were performed to validate the inhibitory effects of the compound. We identified the selective PI3Kβ inhibitor TGX221 as a selective inhibitor for ccRCC with both VHL and SETD2 mutations. TGX221 also targeted cancer cells with CDKN2A and PTEN mutations. Changes in PTEN and CDKN2A gene sets were associated with worsened prognosis of ccRCC. TGX221 substantially and selectively inhibited the down stream products of VHL, SETD2, and PTEN in ccRCC cells with VHL and SETD2 mutations. TGX221 also exhibited significant selectivity in inhibiting cell motility and tumourigenesis of ccRCC cells with VHL and SETD2 mutations. TGX221 is a novel inhibitor with high selectivity for ccRCC with VHL and SETD2 mutations. It also targeted PTEN and CDKN2A mutations. How those genes were associated with PI3Kβ warranted further investigations.

Clear cell renal cell carcinomas (ccRCC) are the predominant subtype of RCC characterized by its chemo-resistant nature and can be distinguished by underlying gene mutations^1^. Mutation in the von Hippel-Lindau (VHL) gene has long been recognized to have close association with the pathogenesis of hereditary or sporadic ccRCC. The resultant upregulation of hypoxia inducible factors (HIF1α and HIF2α, also known as HIF1A and EPAS1) due to failure of ubiquitination by the mutated VHL leads to vast neovasculature, which subsequently promotes tumour growth^2^. Recent reports using novel sequencing techniques have identified inactivation of histone modifying genes, including PBRM1, BAP1, and SETD2, distinguishing a new subtype of ccRCC^3^,^4^,^5^. Despite that some studies have reported the clinical association of those genes, how those genes participate in the carcinogenesis of ccRCC remains mostly unknown.

Current targeted therapies for metastatic ccRCC are mainly tyrosine kinase inhibitors targeting angiogenesis rather than the cancer cell per se, conferring limited effect and intolerance due to major adverse events^6^. Application of rapalogs that targets the mechanistic target of rapamycin (mTOR) of the RCC cells appeared even less promising, prolonging roughly 4 months in overall survival (OS)^7^. Therefore, exploiting novel agents targeting signature mutations in ccRCC in the era of big data and next generation sequencing techniques could not only increase the treatment efficacy but reduce the off-target side effects as well. Several collaborative cancer genomic and genetic studies have provided overwhelmingly profound insights into the cancer characteristics, which had never been revealed in most previous reports with limited sample source and technical supports. However, problems emerged as clinicians could hardly handle the astronomically complexity of the genetic data whilst statisticians had a hard time finding the biological contribution for the associations they found. Therefore, development of public portal or platform for direct visualization of the statistical results could substantially promote the understanding of the disease by medical practitioners. Here, we report using the online analytical tools of two major cancer genetic databases, The Cancer Genome Atlas (TCGA) and Genomics of Drug Sensitivity in Cancer (GDSC) to identify a novel promising compound for the selective inhibition of ccRCC with VHL and SETD2 mutations, and provide clues for the interactions between those common mutations in kidney cancer.

## Results

### RCC cells with both VHL and SETD2 mutations are sensitive to TGX221

**Figure 1:**
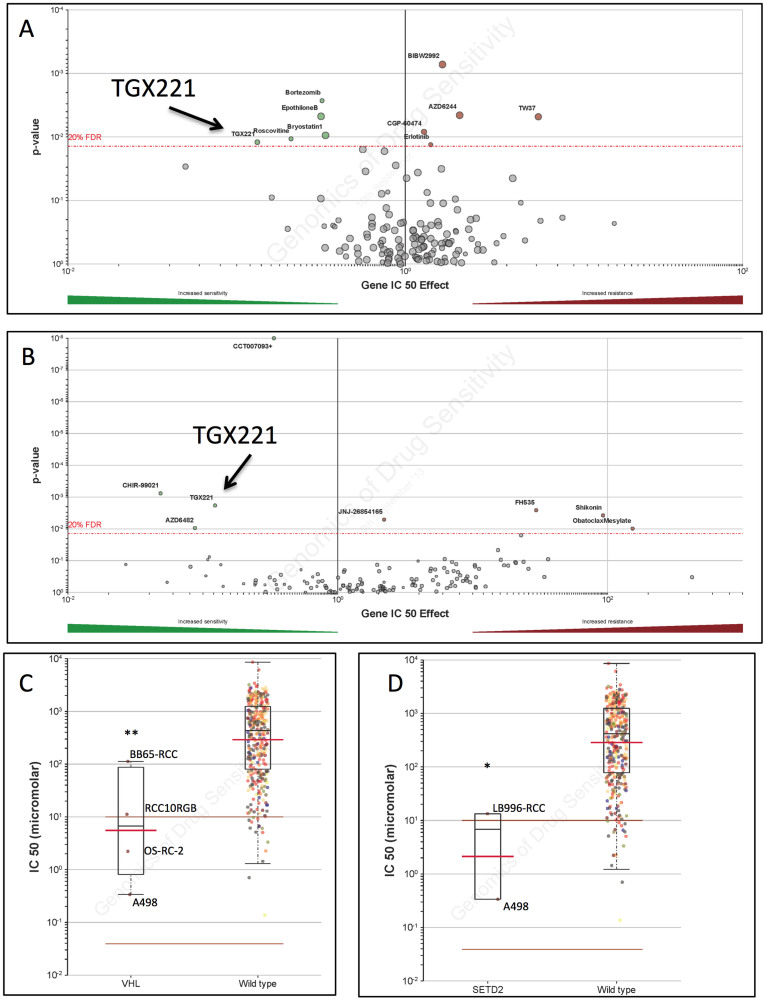
RCC cells with mutated VHL or SETD2 were sensitive to TGX221, reproduced from the Genomics of Drug Sensitivity in Cancer (GDSC) database. Volcano plotting showing compounds marked in green as sensitive in the setting of RCC cells with (A) VHL gene and (B) SETD2 gene mutations, with TGX221 present in both settings. Scattered plotting showing cells with VHL (C) or SETD2 (D) mutations were all RCC cells. (*P <0.05; **P <0.01)

**Figure 2:**
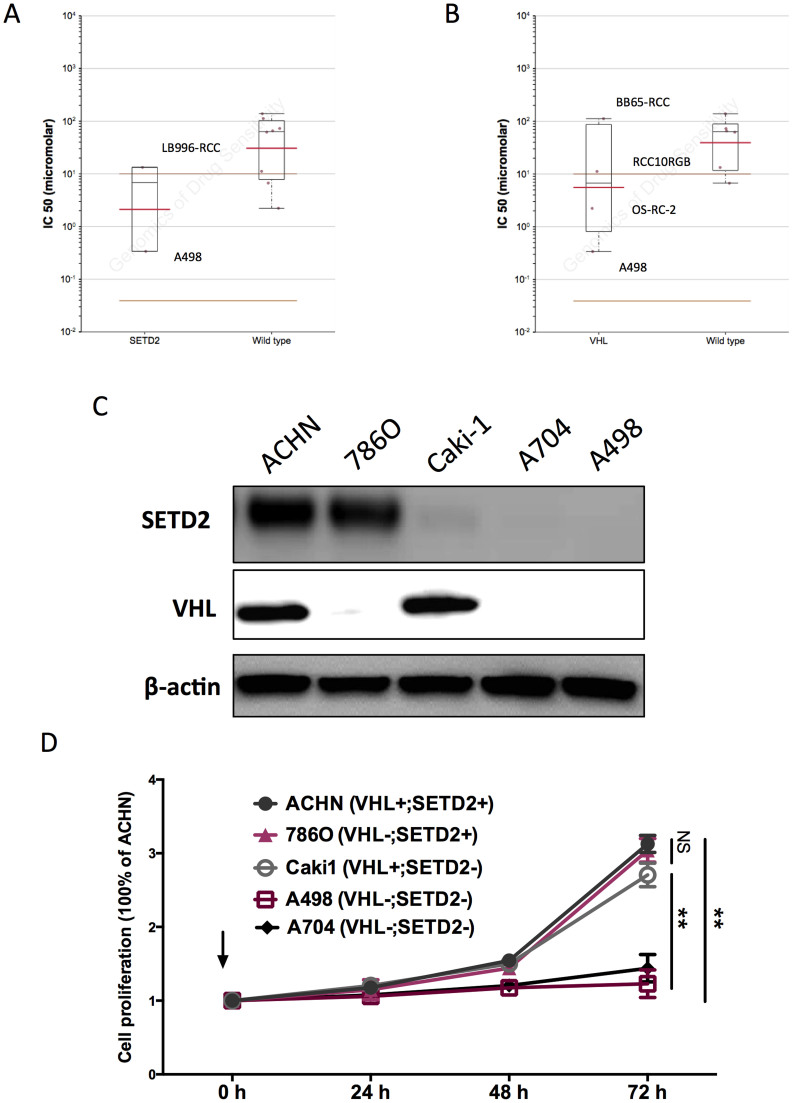
TGX221 selectively inhibited RCC cells with both VHL and SETD2 mutations. Reproduction of Genomics of Drug Sensitivity in Cancer (GDSC) database by excluding cancer of other types showed that RCC cells with (A) SETD2 or (B) VHL mutations was not significantly inhibited by TGX221. Five RCC cell lines were selected for VHL/SEDT2 status validation. (C) A498 and A704 cells harboured mutations in both VHL and SETD2. (D) Proliferation assay using TGX221 at 5 μM showing RCC cells with both VHL and SETD2 mutations were selectively inhibited by TGX221.

Current targeted therapy for metastatic ccRCC conferred limited improvement to survival and could easily induce drug resistance^8^. Also, the first line systemic therapy for ccRCC targeted at neovascularization but not at the tumour cells. With the scope of minimizing off-target effect and exploiting potent tumour inhibitors, we studied the GDSC database to find potential selective compounds. We searched drugs with significant selectivity to commonly mutated genes in ccRCC, including VHL, SETD2, BAP1, and PBRM1. Solely VHL and SETD2 mutations were included in the database analysis. There were 5 hits for VHL and 4 hits for SETD2, among which the TGX221 showed significant selectivity for both VHL and SETD2 mutations (Fig. 1A–B). We then studied the tissue specificity of TGX221 and found that only renal cell carcinoma harbours VHL and/or SETD2 mutations in GDSC database and TGX221 exhibited sensitivity for RCC cells with such mutations (Fig. 1C–D). We then studied TGX221 displayed selectivity within RCC cells with mutations in SETD2 and/or VHL. Further investigation showed that GDSC database did not cover drug sensitivity in all RCC cell lines. Therefore, when only RCC cells were included for drug sensitivity analysis, the selectivity of TGX221 lost statistical power (Fig. 2A–B). By cross-referencing COSMIC database, we summarized all types of RCC in GDSC with mutation status in genes of our interest (Table 1). Further protein blotting confirmed the COSMIC data for mutation status of SETD2 and VHL in each cell line (Fig. 2C). It was noteworthy that some cell lines with SETD2 mutation still showed very weak SETD2 levels (Fig. 2C). We then recapitulated proliferation assay using RCC cell lines with different SETD2 and VHL status. We found that RCC cells with both SETD2 and VHL mutations were selectively inhibited by TGX221 (Fig. 2D). Here we showed that PI3Kβ inhibitor, TGX221 conferred selective inhibition in RCC cells with both SETD2 and VHL mutations.

### Altered PTEN and CDKN2A pathways play critical roles in renal cell carcinoma

**Figure 3:**
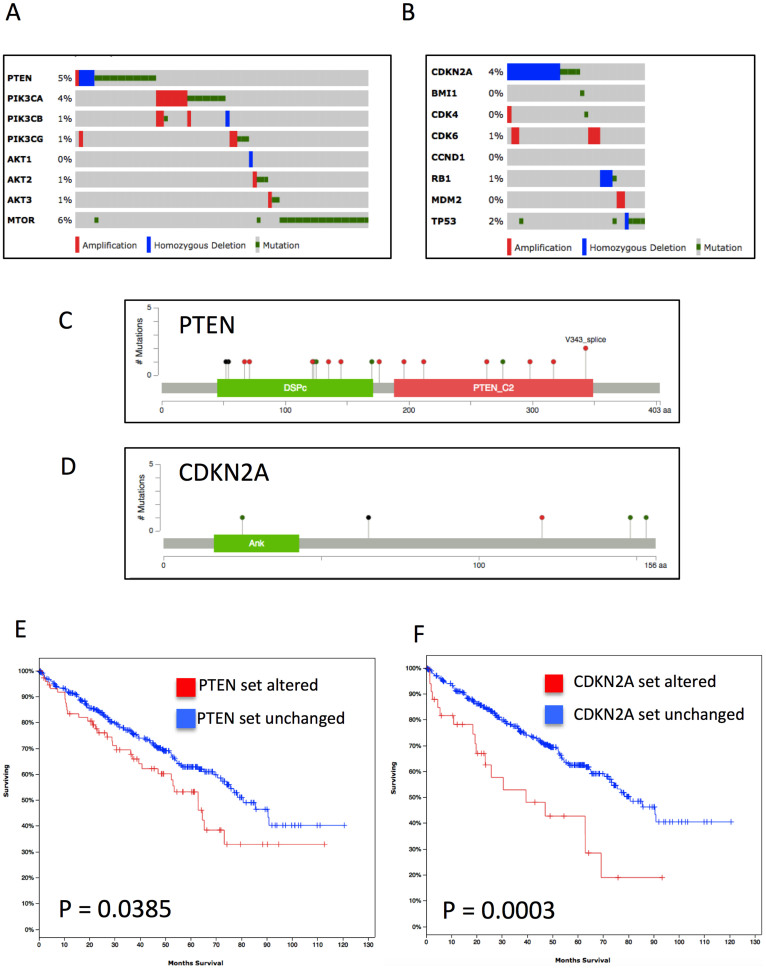
Genes from PTEN and CDKN2A gene sets were frequently mutated in ccRCC. Reproduction of The Cancer Genome Atlas (TCGA) database showing homozygous deletion (blue bars), amplification (red bars), and mutations (green dots) of (A) PTEN and (B) CDKN2A gene sets in ccRCC patients, with only limited mutations in the (C) PTEN and (D) CDKN2A gene per se (Green for missense; Red for truncating; Black for inframe and other; and purple for residues that are affected by different mutation types at the same proportion); Change in (C) PTEN and (D) CDKN2A gene sets were associated with poorer survival.

As aforementioned, off-target effects generated unfavourable events that could often be so intense that treatment was forced to cease^9^. We therefore studied other significant targets of TGX221 throughout all types of cancer cells in GDSC. Besides VHL and SETD2, the compound was also selective for cancer cells with mutation in PTEN and CDKN2A (data not shown). We therefore investigated the mutation and copy number variance (CNV) in PTEN and CDKN2A gene sets. The PTEN and RB gene sets were altered in 18.3% and 8.2% of cases, respectively (Fig. 3A–B), despite the relatively low frequency of PTEN and CDKN2A mutations in ccRCC patients (Fig. 3C–D). We also noticed significant worsened prognosis in patients with altered PTEN or CDKN2A gene sets (Fig. 3E–F). In all, we concluded that although mutation/CNV frequency in PTEN or CDKN2A was low in ccRCC, changes in gene set conferred worsened OS. These data further complemented the selective inhibitory effect of TGX221 in ccRCC.

### Mutated NOTCH1 gene set may confer resistance to TGX221

**Figure 4:**
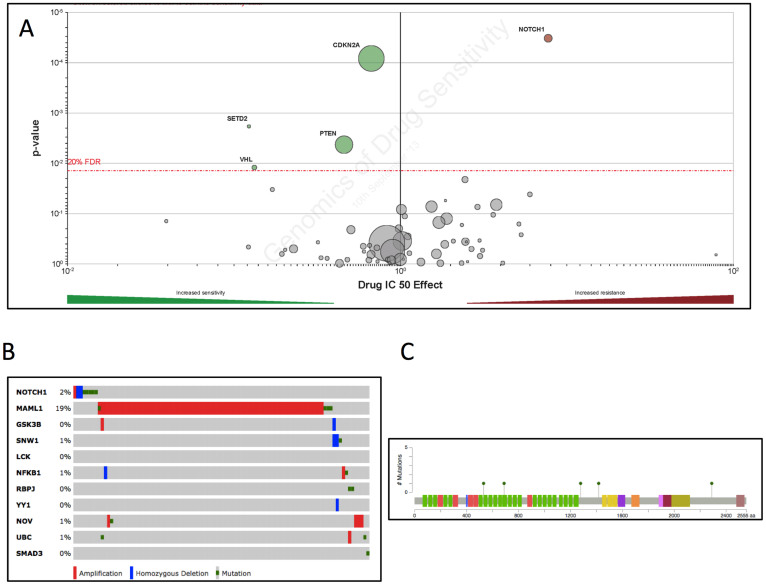
Mutation in NOTCH1 is rare in ccRCC. Reproduction of Genomics of Drug Sensitivity in Cancer (GDSC) database showing (A) NOTCH1 as the only significant resistant mutation in all types of cancer cells; (B) Reproduction of The Cancer Genome Atlas (TCGA) database showing 2% of ccRCC patients harboured NOTCH1 mutations or copy number variance (CNV) whereas amplification of MAML1, a co-activator within NOTCH1 gene set was noticed in ~18% of patients; (C) All mutations were missense mutations.

Mutations that conferred to drug resistance remained a problem for targeted therapy in all types of cancer, regardless of pre-existing or de novo^10^. We found from the GDSC that mutation in NOTCH1 conferred resistance to TGX221 (Fig. 4A). We then analysed TCGA KIRC and found that NOTCH1 mutation/CNV was present in solely 2% of ccRCC patients. All NOTCH1 mutations were missense mutations (Fig. 4C). Interestingly, while most genes within NOTCH1 gene set harbour mutations at a low frequency, we noticed high prevalence of MAML1 amplification in ~19% of patients, possibly due to high amplification of 5q in ccRCC. How mutations in NOTCH1 and its gene set might impact sensitivity of TGX221 warranted further investigation.

### TGX221 selectively inhibits tumourigenesis of RCC with mutated VHL or SETD2

**Figure 5:**
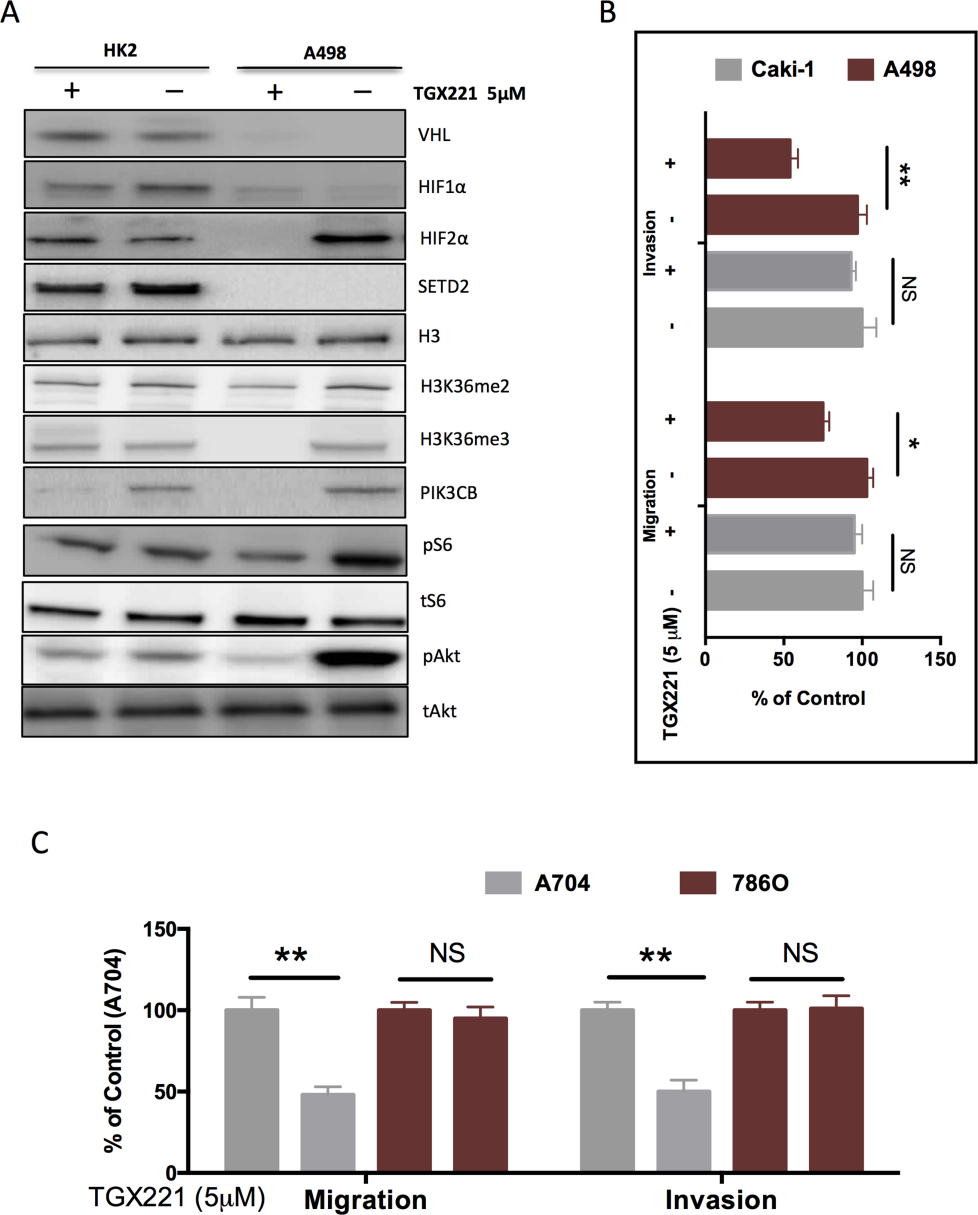
TGX221 selectively inhibits cell motility of RCC with mutated VHL and SETD2. (A) Application of TGX221 in VHL- and SETD2-wildtype HK2 cells and in both mutated A498 RCC cells showing selective inhibition of downstream effectors of VHL, SETD2, and PTEN in A498 cells; (B) Compared with Caki-1 RCC cells that harboured wildtype VHL, TGX221 selectively inhibited the invasiveness, and migration in A498 cells; (C) Compared with 786O RCC cells that harboured wildtype SETD2, TGX221 selectively inhibited the invasiveness, and migration in A704 cells (*P <0.05; **P <0.01).

**Figure 6:**
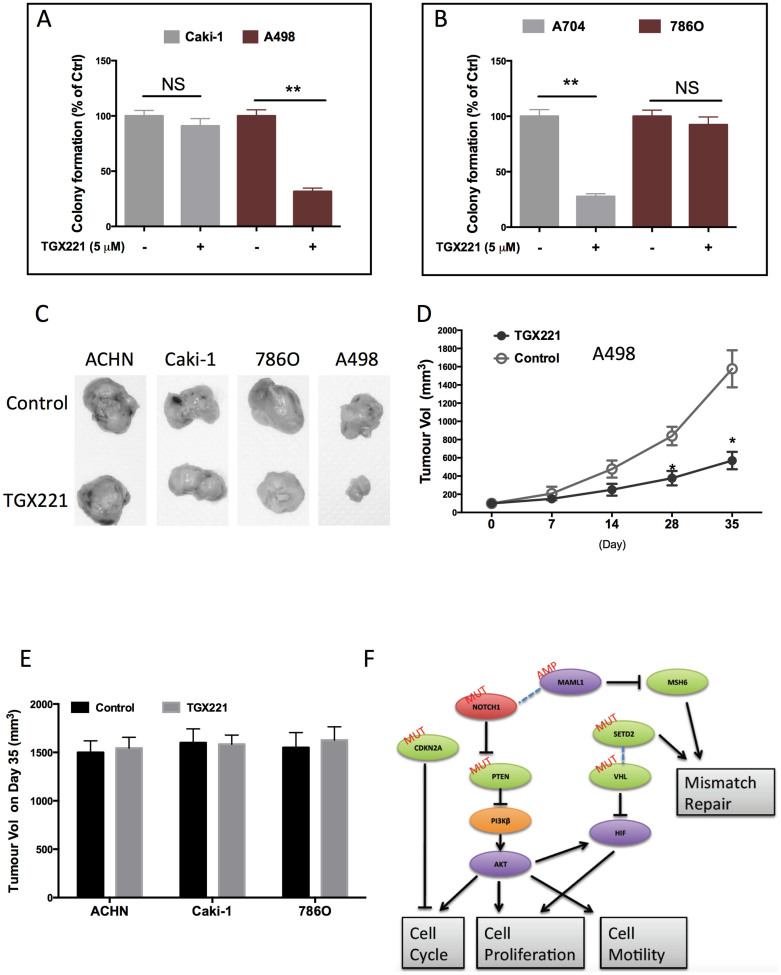
TGX221 selectively inhibits tumourigenesis of RCC with mutated VHL and SETD2. (A) Compared with Caki-1 RCC cells that harboured wildtype VHL, TGX221 selectively inhibited the colony formation in A498 cells; (B) Compared with 786O RCC cells that harboured wildtype SETD2, TGX221 selectively inhibited the colony formation in A704 cells; (C) Xenograft tumour model using RCC cells with different VHL and SETD2 status showing selective inhibition in tumour growth by TGX221, (D) which significantly inhibited A498 tumour growth and conferred insignificant inhibition in ACHN, 786O, or Caki-1 tumours; (E) Schematic diagram of how TGX221 could linked multiple pathways/gene sets in ccRCC (*P <0.05; **P <0.01).

As per our results computed from 2 solid databases, we further studied the effect of TGX221 on kidney cells with different genetic background. We found that A498 cells did not express HIF1α but expressed HIF2α instead (Fig. 5A), in accordance with previous reports^11^. TGX221 almost completely abolished the HIF2α in A498 cells but had minimal effect on either HIF1α or HIF2α in HK2 normal renal tubule epithelial cells (Fig. 5A). Of note, TGX221 substantially inhibited the trimethylation of H3 in the SETD2 mutated scenario (Fig. 5A), which was in reminiscence of the similar results in leukaemia^12^. As a selective PI3Kβ inhibitor, TGX221 abolished PI3K in both cell lines and strongly inhibited phosphorylation of Akt at Ser473 (Fig. 5A). Nonetheless, the mTOR activity indicated by phosphorylation of S6 was not apparently altered with the treatment of TGX221. In order to compare the selectivity of TGX221 in RCC cells with different genetic basis, we used Caki-1 ccRCC cell line, which was known to have wildtype VHL and mutated SETD2^4^,^11^. Like in proliferation assays, TGX221 demonstrated strong selectivity for A498 cells in comparison to Caki-1. The inhibitory effect of TGX221 in both migration and invasiveness were significant in A498 cell whereas in Caki-1, the inhibition was insignificant (Fig. 5B). These results were recapitulated between SETD2/VHL mutated RCC cells and SETD2-WT 786O cells (Fig. 5C). We then studied the in vitro tumourigenesis assay and found that RCC4 cells with both SETD2 and VHL mutation was selectively inhibited by TGX221 (Fig. 6A–B). When tumourigenesis in xenograft model was studied, we found that TGX221 significantly inhibited growth of A498 cells (Fig. 6C–D). Although tumours with either mutation in SETD2 or VHL, and both WT status did not change in size following TGX221 treatment (Fig. 6D), ACHN tumours were in general slightly larger than Caki-1 or 786O tumours (Fig. 6C). Together, we used in vitro motility assays and in vivo models to confirm that TGX221 selectively inhibited RCC cells with VHL and SETD2 mutations and conferred inhibition to tumourigenesis of RCC with mutated target genes.

## Discussion

In the current study, we have used 2 major databases to identify a compound, TGX221 not previously studied in ccRCC that has strong selectivity for tumours having VHL, SETD2, PTEN and CDKN2A mutations. Distinctive from anti-angiogenic agents that target the neovasculature resultant from uncurbed HIF expression in VHL mutated cases, compound that targets the VHL mutated tumour cell itself appears intriguing. As the major downstream effector when VHL is mutated, HIF2α is now suggested to play more critical role in RCC than its isoform HIF1α to induce vascular endothelial growth factor (VEGF)^11^. HIF2α is also believed to complement HIF1α's function in cells that do not express HIF1α, like A498 in our study, and in the context of VHL-wildtype RCCs. Therefore, HIFs serve more of a validation role in our study than elucidating the drug-gene interactions. Though targeting VHL in RCC cells with peptide vaccine has been reported^13^, how TGX221 confers the selectivity is mostly unclear. Even more confounding is the selectivity for SETD2 mutations of TGX221 in RCC. Amongst the 4 most common gene mutations located on 3p, SETD2 together with PBRM1 and BAP1 has merely been identified recently. SETD2 functions in the epigenetic regulation of a series of gene expression in, but not limited to RCC^14^,^15^. In another study that also reproduced TCGA KIRC data, SETD2 is associated with worse cancer specific survival (CSS)^15^, a statement that still warrants further validation. Lack of insight of the biological effect of SETD2 solely entitles it as a novel disease marker and less of a treatment target in ccRCC. To date, there have not been reports on the targeted therapy for SETD2 in any cancer type, let alone RCC. Therefore, the selectivity of TGX221 for both VHL and SETD2 implies an inherent connection between those common mutations in a variety of cancers. More specifically in our case, only RCC cells with both mutations show significant sensitivity than RCC with other genetic basis. This reminds of a recent study showing that only when VHL and BAP1 are simultaneously deleted, can mice develop ccRCC, solving a long lasting problem in animal model establishment^16^. We therefore speculate that high prevalence of VHL silencing in ccRCC could play different or partially different roles in the presence of co-loss of other commonly mutated genes, as in our case, SETD2. Nonetheless, the TCGA study using exome sequencing fails to capture many of VHL deletions due to the high GC content within its first exon and therefore yields a detection rate of VHL mutation/deletion at ~54%^17^. In general, VHL is believed to be inactivated in 80 ~ 90% of ccRCC^18^, among which allelic deletion or loss of heterozygosity takes up>90%^19^, gene mutation takes up 50%^20^, and promoter hypermethylation takes up 10%^21^. In a similar comprehensive study depicting genetic landscape of ccRCC by Sato Y et al, SETD2 and BAP1 mutations are likely to be acquired and selected from within pre-existing VHL- and/or PBRM1-mutated clones and contribute to tumour progression^18^.

Besides the selectivity for VHL and SETD2, TGX221 also targets CDKN2A and PTEN mutations that, albeit not unique in ccRCC, play critical roles in a variety of cancers^22^,^23^. Selective PI3Kβ inhibitors, like TGX221 and KIN193 are known to be sensitive in PTEN mutated cells and are thought to minimize the side effects caused by pan-PI3K inhibitors and p110α-selective inhibitors^24^,^25^. It is thus intriguing that PTEN is merely mutated/deleted in 5% of ccRCC patients^17^ whereas TGX221 has not been reported to be selective to other mutations. Also, mutation in PIK3CB was only detected in 1 patient in TCGA KIRC, in contrast to the commonly altered PI3K pathway in which PIK3CA had high-level amplification^3^. Besides loss of 3p and gain of 5q that were validated in the KIRC series^17^, it has recently been found that 9p deletion, encompassing CDKN2A, is associated with higher stage, larger tumours, necrosis, microvascular and renal vein invasion, and higher SSIGN (stage, size, grade and necrosis) score^26^. Patients with 9p-deleted ccRCC were at a higher risk of recurrence and RCC-specific mortality^26^. As a tumour suppressor, CDKN2A encodes 2 proteins that participate in two pivotal cell cycle regulatory pathways: the p53 pathway and RB pathway, and in our study, we focused on the contribution of altered RB gene set in ccRCC.

Given that those seemingly independent genes are related by the high selectivity of TGX221, we speculate that these pathways could converge at certain point, a phenomenon commonly occurring in RCC^27^. We have summarized significant changes in phosphorylation in ccRCC with VHL or SETD2 mutation in KIRC and have found that alteration in mTOR, MAPK, and cell cycle related proteins are most common in both settings implying a potential linkage between the two genotypes in ccRCC (Table 2). Combining the established interactions for all the target genes in our study, we speculate that AKT could stand as the hub of downstream effector that is targeted by TGX221 in ccRCC, which leads to curbed cell cycle and proliferation (Fig. 6F). All these speculation however still warrant insightful studies to elucidate both the pharmacological mechanisms of TGX221, and the interplays between common genetic and genomic variances in kidney cancer.

## Conclusion

CcRCCs have been characterized with frequent mutations in the VHL and SETD2. We have found via GDSC database that TGX221 is a selective inhibitor for RCC cells with VHL and SETD2 mutation. We have also found TGX221 targeted PTEN and CDKN2A mutation of which the pathway alteration impact the survival of ccRCC patients. TGX221 significantly inhibited tumourigenesis of ccRCC, yet how those target genes interact warrants further investigation.

## Methods

### Data mining and analysis of TCGA database

As the initial TCGA global analysis on ccRCC was published^17^ and as per the latest publication guidelines of TCGA^31^, our reproduction of the data had no limitations or restrictions. The TCGA kidney renal clear cell carcinoma (TCGA KIRC provisional) database analysed on the cBioPortal platform contained in all 522 renal tumours of clear cell type with one or more of the following somatic data: mutations; putative copy-number alterations; mRNA expression data presented by Z-scores detected with microarray, with RNA-seq, or the mRNA/miRNA expression within all genes; and protein/phosphoprotein level detected with reverse phase protein array (RPPA)^28^,^29^. As we intended to study the relations between mutations and clinical characteristics, case set within “All Complete Tumours” or “Tumours with sequencing and CNA data” were selected, in which the tumour and clinical profiles were all complete in all 413 patients, whereas mutation and CNV was complete in 415 patients. “Protein change” was used to compare protein levels between different statuses of query genes. “Survival” was used to plot Kaplan-Meier curves and “Network” was used to plot computed gene interactions.

### Data mining and analysis of the GDSC database

The GDSC project is a collaboration between the Cancer Genome Project at the Wellcome Trust Sanger Institute (UK) and the Center for Molecular Therapeutics, Massachusetts General Hospital Cancer Center (USA), and is funded by the Wellcome Trust^30^. We confirm that reproduction and publication of the GDSC data and figures complied with the organization. The complexity of the data encompassed the sensitivities and genomic profiles of a variety of cancer cell lines to a number of compounds. To facilitate the visualization of the astronomical data matrix, the public online platform was developed with all data downloadable and plots reproducible^31^. We started by searching compounds with significant selectivity for VHL mutations. We then tried to narrow the hits by crosschecking whether other targets of the drugs conferred a “second” selectivity to the renal cancer cells. The volcano plots, elastic nets, scatter plots, and the Mann-Whitney-Wilcoxon (MWW) tests were generated and computed via the GDSC online platform.

### Cell culture

The A498, 786O, A704, ACHN, Caki-1 renal cancer cells, and HK2 normal renal tubule epithelial cells were acquired from the American Type Culture Collection (ATCC) and were cultured with RPMI-1640 media with 1 mg/ml of 80% bovine insulin, and 20% foetal bovine serum. TGX221 was purchased from Selleckchem. Dimethyl sulfoxide (DMSO) was a product of Sigma-Aldrich.

### Western blotting

Blotting was performed as per our previous reports^32^,^33^. Briefly, total protein of lysates was extracted and equal amount of protein was loaded onto 10% sodium dodecyl sulphate polyacrylamide gel for electrophoresis. Gels were subsequently transferred to nitrocellulose membrane. The membranes were blockaded for 1 h with 5% non-fat milk. Primary antibodies against VHL (Abcam), HIF1α (Abcam), HIF2α (Abcam), SETD2 (Abnova), H3 (Abcam), H3K36me2 (Lys36, Cell Signaling), H3K36me3 (Lys36, Cell Signaling), PI3 Kinase p110β (Cell Signaling), pS6 (Ser235/236, Cell Signaling), total S6 (Cell Signaling), pAkt (Ser473, Cell Signaling) and total Akt (Cell Signaling) were then added and membranes were kept incubating at 4°C overnight. Corresponding secondary antibodies were applied followed by electrochemiluminescence (ECL) processing.

### Migration and invasion assay

In vitro migration and invasion assays were performed according to standardized protocol^34^. Briefly, RCC cells were seeded in the top chamber of the 8.0 μm pore size cell culture inserts that were either coated or uncoated with matrigel for migration and invasion assays, respectively. Then the inserts were placed in a 24-well plate filled with medium with 5% foetal bovine serum. Cells that penetrated to the underside surfaces of the inserts were fixed and stained with the Diff-Quick (Fisher Scientific) method and were counted under the microscope. The mean of cell number of three high power fields for each condition was calculated. Assay was done in triplicates.

### Colony formation assay

Colony formation study was performed using soft agar assay. Briefly, 1,000 Caki-1 or A498 cells were resuspended in medium containing 10% FBS with 0.4% agarose, which was layered on the top of 0.6% agar in medium supplemented with 20% FBS on 60-mm plates. After 2 weeks of culture at 37°C, plates were stained with 0.005% of crystal violet for 1 h. Colonies were counted microscopically and the relative colony numbers were measured.

### RCC xenograft models

Forty-eight male BALB/c athymic nude mice at 6 weeks of age were bred in SPF (special pathogen-free) grade laboratory. Mice were randomly divided into 8 groups (ACHN, 786O, A498, and Caki-1; Treatment versus control). A total of 1.5 × 10^7^ cancer cells resuspended in 100 μl of PBS were injected subcutaneously at the left axilla of each mouse. Tumours became perceptible at approximately 4 mm in diameter on approximately day 7. Thereafter, intratumoural injection of TGX221 at 1 mg/kg or control (PBS) at equivalent volume was given every 3 days. All mice were sacrificed on Day 35 and tumours were extracted. Tumour size was calculated with the formula, Length × Width^2^ × 0.5236. All animal experiments were performed in accordance with relevant guidelines and regulations of Department of Laboratory Animal Science of Fudan University with the licensing No. SYXK(SH)2009-0082. All experimental protocols were approved by Institutional Review Board of Department of Laboratory Animal Science of Fudan University.

### Statistical analysis

Comparisons between groups were analysed with the 2-tailed Student's *t*-test. The P value of <0.05 was accepted as statistically significant.

## Figures and Tables

**Table 1:** Kidney cells and mutation status in GDSC database

Cell line	COSMIC	Cell type	Mutation					IC50 (Log10)
			VHL	SETD2	BAP1	PBRM1	NOTCH1	
769-P	910922	Clear cell renal cell carcinoma	*		*			
786-0	905947	Clear cell renal cell carcinoma	*					
A498	905948	Clear cell renal cell carcinoma	*	*				−1.085304
A704	905948	Renal cell adenocarcinoma	*	*				
ACHN	905950	Clear cell renal cell carcinoma				*		
BB65-RCC	753533	Renal cell adenocarcinoma	*					4.721833
BFTC-909	910698	Transitional cell carcinoma				*		
CAKI-1	905963	Clear cell renal cell carcinoma		*				
CAL-54	910952	Renal cell carcinoma						
G-401	907299	Renal rhabdoid tumour						
LB1047-RCC	753577	Renal cell carcinoma				*		4.183081
LB2241-RCC	753578	Renal cell carcinoma						4.125849
LB996-RCC	753585	Renal cell carcinoma		*				2.589532
OS-RC-2	909250	Clear cell renal cell carcinoma	*			*		0.800791
RCC10RGB	909974	Renal cell carcinoma	*		*			2.415331
RXF393	905978	Hypernephroma						1.904166
SK-NEP-1	909730	Wilm's Tumour						4.933583
SN12C	905979	Renal cell carcinoma			*			
TK10	905980	Renal cell carcinoma						4.287613
U031	905981	Renal cell carcinoma						
VMRC-RCZ	909781	Renal cell carcinoma	*					

**Table 2:** Changes in phosphorylation of proteins in renal clear cell carcinoma with VHL or SETD2 mutation, computed using TCGA KIRC data

Mut. Gene	Tumourigenesis	Target		Average abundance		p-value	Signature
		Protein	Residue	Unaltered	Altered		
**VHL**	anti	**CDKN1B**	pT157	0.18	−0.18	0.0004	Cell cycle
	anti	**CHEK1**	pS345	0.12	−0.19	0.0007	Cell cycle
	pro	**RPS6KA1**	pT359	0.17	−0.07	0.0160	mTOR
	pro	**EIF4EBP1**	pT70	0.14	−0.08	0.0360	mTOR
	anti	**RB1**	pS807	0.09	−0.1	0.0490	Cell cycle
	pro	**EGFR**	pY1173	−0.11	0.17	0.0060	EGFR
	pro	**EGFR**	pY1068	−0.08	0.17	0.0120	EGFR
**SETD2**	pro	**EIF4EBP1**	pS65	0.11	−0.44	0.0000	mTOR
	pro	**PRKCA**	pS657	−0.08	−0.43	0.0005	MAPK
	anti	**CHEK2**	pT68	0.06	−0.43	0.0020	Cell cycle
	pro	**PRKCD**	pS664	−0.08	0.29	0.0120	MAPK
	pro	**YBX1**	pS102	0.01	0.32	0.0350	EGFR
	pro	**JUN**	pS73	0.09	−0.19	0.0390	MAPK
	pro	**RAF1**	pS338	0.03	−0.24	0.0410	MAPK
	pro	**RPS6KB1**	pT389	0.02	−0.19	0.0430	mTOR
